# Using Social Media Data to Identify Potential Candidates for Drug Repurposing: A Feasibility Study

**DOI:** 10.2196/resprot.5621

**Published:** 2016-06-16

**Authors:** Majid Rastegar-Mojarad, Hongfang Liu, Priya Nambisan

**Affiliations:** ^1^ Division of Biomedical Statistics and Informatics Mayo Clinic Rochester, MN United States; ^2^ Department of Health Informatics and Administration UW-Milwaukee Milwaukee, WI United States

**Keywords:** social media, drug repurposing, natural language processing, patient comments

## Abstract

**Background:**

Drug repurposing (defined as discovering new indications for existing drugs) could play a significant role in drug development, especially considering the declining success rates of developing novel drugs. Typically, new indications for existing medications are identified by accident. However, new technologies and a large number of available resources enable the development of systematic approaches to identify and validate drug-repurposing candidates. Patients today report their experiences with medications on social media and reveal side effects as well as beneficial effects of those medications.

**Objective:**

Our aim was to assess the feasibility of using patient reviews from social media to identify potential candidates for drug repurposing.

**Methods:**

We retrieved patient reviews of 180 medications from an online forum, WebMD. Using dictionary-based and machine learning approaches, we identified disease names in the reviews. Several publicly available resources were used to exclude comments containing known indications and adverse drug effects. After manually reviewing some of the remaining comments, we implemented a rule-based system to identify beneficial effects.

**Results:**

The dictionary-based system and machine learning system identified 2178 and 6171 disease names respectively in 64,616 patient comments. We provided a list of 10 common patterns that patients used to report any beneficial effects or uses of medication. After manually reviewing the comments tagged by our rule-based system, we identified five potential drug repurposing candidates.

**Conclusions:**

To our knowledge, this is the first study to consider using social media data to identify drug-repurposing candidates. We found that even a rule-based system, with a limited number of rules, could identify beneficial effect mentions in patient comments. Our preliminary study shows that social media has the potential to be used in drug repurposing.

## Introduction

New drug development costs US $500 million to $2 billion and takes 10-15 years [1]. A well-known approach to reduce risk and cost of new drug development is drug repurposing (or drug repositioning) [2]. Drug repurposing (defined as discovering new indications for existing drugs) could play a significant role in drug development, considering the declining success rates of developing novel drugs. From 2007-2009, 30-40% of newly approved drugs were repurposed medications [3]. Considering the high cost of launching a new drug, this emphasis on repurposing could markedly affect drug development. Typically, a new indication for an available drug is identified by chance. However, new technologies and a large number of available resources enable the development of systematic approaches to identify and validate drug repurposing candidates with considerably lower costs. Drug repurposing has been exhaustively studied, and various approaches have been used [3-5] to identify novel drug repurposing candidates, using clinical data [6], genetic information [7-9], and scientific literature [10-13].

Grau and Serbedzija [4] named two types of drug repurposing: (1) identification of off-target drug actions and (2) identification of relevance of a known drug target to a new disease. From an informatics perspective, freely available and relevant resources such as scientific literature, clinical trials, and biological resources can be used to conduct drug-repurposing studies. The compound database PubChem [14] has been used in several drug-repurposing studies [15]. Hoehndorf et al [16] implemented a system that inferred novel associations between drugs and diseases by linking drug-gene associations in the PharmGKB database to phenotype studies and animal models of disease. Moriaud et al [17] presented a computational method that mined the Protein Data Bank [18] to identify drug repositioning candidates. Several studies [10,11,16,17,19,20] considered literature mining for drug repurposing; this approach has been comprehensively reviewed elsewhere [21,22].

Social media provides a platform for patients to share their experiences with illnesses, medications, and also medical centers [23]. Patient posts, usually written in an informal language, contain hidden and valuable information. Owing to the massive amount of data derived from social media, computerized systems are needed to analyze and extract useful information from patient experience. Unlike scientific literature, these comments are usually written by non-experts users who do not have any obligation to follow proper grammar in their comments or report accurate observation. These differences make mining social media more complicated and challenging compared to scientific literature. Nevertheless, there have been several attempts to extract knowledge from social media. Leaman et al [24] examined comments posted in a medical forum to identify reported adverse drug events. After manually annotating a corpus of patient posts, they used natural language processing methods to develop a system that extracted adverse drug reactions from the text. Chee et al [25] studied patient posts on Health and Wellness Yahoo! groups and applied common natural language processing methods to predict adverse drug events and identify medications that might require further scrutiny by the Food and Drug Administration. Freifeld et al [26] evaluated the correlation between adverse drug events reported in Twitter (where statements are limited to 140 characters) and spontaneous reports received by a regulatory agency. Rastegar et al [27] implemented a binary classifier to identify adverse drug reactions in tweets. Sharif et al [28] proposed a sentiment classification framework to detect adverse drug reactions in medical blogs and forums. Recently, Karimi et al [29] provided a corpus of 1321 medical forum posts on patient-reported adverse drug events, which allows researchers to develop and evaluate pharmacovigilance systems.

Although patients mostly use medically oriented social media to describe adverse events associated with drugs [30,31], their experiences may help others to conceive of new indications for existing medications if their descriptions also include beneficial effects. A well-known example is Zolpidem, an insomnia medication that, through social media and patient reviews, was subsequently used for brain injury [32]. Leaman at el [24] identified 157 beneficial effects, in 3600 patient posts that could lead to drug repurposing. The accuracy of these reported beneficial effects in social media may be questionable, but considering the value of drug repurposing and huge amount of available social media data, it is worthwhile to study this type of information and investigate the possibility of identifying potential drug-repurposing candidates. In this study, we assessed the feasibility of using social media data in identifying potential drug repurposing candidates. Our hypothesis is that this imperfect resource could lead to drug repurposing.

## Methods

### Data Sources

In this study, we used data from four public resources: WebMD [33], DrugBank [34], SIDe Effect Resource (SIDER) [35], and Unified Medical Language System (UMLS) [36]. Below are brief descriptions of the resources and their uses in this research.

WebMD is an US corporation that provides Web-based health-related services, including a forum for patients to share their experiences with medications. The comments are entered as free text, and the length of comments is not subject to a character or word count limit. WebMD [33] allows users to score three different aspects of the medication in their reviews: (1) effectiveness, (2) ease of use, and (3) satisfaction. WebMD provides some basic information about the users such as age, sex, and duration of treatment. The patient comments from WebMD were the main material used in this study.

DrugBank is a bioinformatics and cheminformatics resource that provides drug information, such as indication, synonyms, gene target, drug interactions, and structure. This database was used to identify known indications of drugs.

SIDER, developed by Kuhn et al [35], contains information about 1430 marketed medications and 5880 side effects (140,064 drug-side effect pairs) extracted from public documents and package inserts. SIDER retrieved adverse drug reaction and disease names from UMLS to generate a dictionary of side effects. We used SIDER to detect known side effects of drugs mentioned in the comments.

UMLS [36] integrates medical terminology and coding standards to help researchers and developers create interoperable biomedical information systems. We used UMLS resources to create a dictionary of disease names. The dictionary contains all spelling variants of diseases provided in UMLS. The dictionary includes 239,227 entries for 86,839 unique diseases.

### Method

In the first step, we generated a list of the top 180 most frequently searched medications on WebMD. All patient comments pertaining to these drugs were retrieved. Through DrugBank, we collected known and approved indications related to those medications. To locate the drugs in DrugBank, we searched synonyms and brand name entries in addition to drug name entry. In the next step, a list of known side effects for each drug is retrieved from SIDER.

We next developed a natural language processing system to identify beneficial or adverse effects. Any mention of disorders in the reviews was tagged by using two disease named entity recognition (NER) approaches: (1) dictionary-based and (2) machine learning. In the dictionary-based approach, a list of disease names from UMLS was retrieved and a string-matching technique was applied to identify diseases mentioned in the comments. The dictionary-based approach did not consider any grammatical or semantic reasoning or spelling errors. For the machine-learning NER approach, we used MetaMap [37], a tool to recognize UMLS concepts (eg, diseases) in the text. Unlike the dictionary-based method, MetaMap uses natural language processing and computational linguistic techniques to incorporate semantic and grammatical reasoning in the identification task.

We discarded comments that contained only known indications or adverse effects for related medication. We then manually reviewed some of the remaining comments to develop a list of textual patterns commonly used to report beneficial effects or indications. We developed a rule-based system to tag the comments containing at least one of those patterns. In the final step, the tagged comments were manually reviewed to identify potential drug repurposing candidates.

## Results

We retrieved 64,616 patient posts from the top 180 most commonly searched drugs in WebMD (mean number of posts per drug was 358). Lisinopril (an angiotensin-converting enzyme inhibitor used to treat high blood pressure and heart failure) had the most comments (n=2931), whereas metoclopramide (used to treat gastric esophageal reflux disease) had the fewest comments (n=8). Table 1 shows the top 10 reviewed medications and includes the three most frequently named diseases in the respective comments.

The dictionary-based NER approach identified 2178 disease names in the comments, whereas MetaMap identified 6171 disease mentions. Table 2 shows the 10 most commonly named diseases in the comments (after disambiguated terms were removed manually). Of the 180 drugs, 164 (91.1%) were listed in DrugBank but only 74 (41.1%) were listed in SIDER. We filtered comments to remove text describing known indications and adverse drug events from the list of recognized disease names; frequently named diseases from the text that remained are shown in Table 3 (note the overlap with Table 1).

**Table 1:** Most-reviewed medications in WebMD and most frequently named diseases in the reviews.

Drug name	Reviews, n	Disease names, n		Most frequent disease names	
		Dictionary-based	MetaMap	Dictionary-based	MetaMap
Lisinopril	2931	288	1135	Itch	Blood pressure
				High blood pressure	Cough
				Rash	Dry cough
Hydrocodone-acetaminophen	2684	320	987	Arthritis	Pain
				Itch	Back pain
				Chronic pain	Arthritis
Phentermine	1931	207	860	Dry mouth	Dry mouth
				Depression	Weight loss
				Obese	Blood pressure
Cymbalta	1651	320	1063	Depression	Depression
				Itch	Anxiety
				Fibromyalgia	Weight gain
Lexapro	1609	269	864	Depression	Depression
				Itch	Weight gain
				Panic attack	Anxiety
Effexor	1568	290	943	Depression	Depression
				Itch	Dizziness
				Panic attack	Anxiety
Tramadol	1404	261	826	Arthritis	Pain
				Fibromyalgia	Back pain
				Migraine	Dizziness
Trazodone	1305	226	701	Depression	Insomnia
				Dry mouth	Depression
				Chronic insomnia	Anxiety
Topamax	1191	271	840	Migraine	Migraine
				Gist	Headaches
				Memory loss	Tingling
Percocet	1125	245	713	Itch	Pain
				Chronic pain	Abuse
				Arthritis	Back pain

**Table 2:** Most frequently named diseases in reviews.

Dictionary-based		MetaMap	
Disease	Count	Disease	Count
Depression	5602	Pain	9990
Itch	3594	Depression	4921
Migraine	1610	Blood pressure	4016
Dry mouth	1269	Weight gain	3778
Infection	1218	Dizziness	3484
Panic attack	1174	Anxiety	3323
Rash	1086	Headache	2216
Arthritis	905	Nausea	1977
Fibromyalgia	850	Relief	1671
Mood swing	730	Dry mouth	1279

**Table 3:** Most-reviewed medications in WebMD and most frequently named diseases in the reviews after removing known indications and adverse drug events.

Drug name	Disease names, n		Most frequent disease names	
	Dictionary-based	MetaMap	Dictionary-based	MetaMap
Lisinopril	280	1124	Itch	Blood pressure
			High blood pressure	Cough
			Rash	Dry cough
Hydrocodone-acetaminophen	320	987	Arthritis	Pain
			Itch	Back pain
			Chronic pain	Arthritis
Phentermine	195	834	Depression	Weight loss
			Obese	Blood pressure
			High blood pressure	Sleeping
Cymbalta	320	1063	Depression	Depression
			Itch	Anxiety
			Fibromyalgia	Weight gain
Lexapro	269	864	Depression	Depression
			Itch	Weight gain
			Panic attack	Anxiety
Effexor	290	943	Depression	Depression
			Itch	Dizziness
			Panic attack	Anxiety
Tramadol	200	670	Fibromyalgia	Pain
			Chronic pain	Back pain
			Migraine	Headache
Trazodone	196	609	Chronic insomnia	Depression
			Migraine	Anxiety
			Fibromyalgia	Headache
Topamax	271	840	Migraine	Migraine
			Gist	Headaches
			Memory loss	Tingling
Percocet	245	713	Itch	Pain
			Chronic pain	Abuse
			Arthritis	Back pain

### Textual Patterns

The frequency of ten common textual patterns, used to report beneficial effects, were counted in the comments and shown in Table 4. Table 5 shows the frequency of the patterns after removing the comments, which mentioned only known side effects or indication. A manual review of the remaining comments identified five drugs with potential for repurposing (see Table 6).

**Table 4:** Textual patterns to identify drug-repurposing candidates.

Pattern	Count	Example drugs and comments^a^
I use * for	307	Methadone: I use this for diabetic neuopathy. works well with very little side effects.
		Percocet: I use this for M.S. pain
		Percocet: I use this med for peripheral neuropathy pain.
I use it for	42	Cymbalta: My use of Cymbalta is two fold. I use it for depression and fibromyalgia pain.
		Spironolactone: I use it for acne. Go figure it works
		Promethazine: I use it for gastroparesis. I also use it for sleep 4 or 5 times a month
It helps with	131	Nucynta: It helps with my pain from surgery
		Percocet: it helps with my back pain, better then any drug
		Klonopin: I like this medication it helps with my anxiety.
It help with	11	OxyContin: it help with muscle spasms
		Neurontin: i had drop foot and much pain. it help with the pain along with the 3 epidurals i receiveed in my spine.
		Cymbalta: i started this medication years ago. not only did it help my depression, it help with my auto immune, muscle and nerve pain.
I take it	1,161	Nucynta: I take it for severe headache and neck pain from arthritis, bulging disks, and bone spur in my neck (cervical spine)
		Methadone: I take it for chronic pain it helps a lot
		Flexeril: I take it for muscle spasms related to fibromyalgia.
I take it for	91	Methadone: I take it for chronic pain it helps a lot
		Methadone: I take it for degenertive disk deteration in my neck.
		Hydrocodone-acetaminophen: i take it for my scholiosis of my back
It works for	258	Methocarbamol: It works for my muscle tension, but gives me a headache.
		Diazepam: it works for my pain weal good
		Tramadol: It works for my Arthritis Pain.
It is useful for	0	…
Useful for	18	Methadone: very useful for chronic and severe pain associated with fibromyalgia/rheumatoid arthritis.
		Effexor: I have been reading the reviews of this med. I have been using it for 1.5 yrs and has been very useful for my depression.
		Ultram: this med has been very useful for my hip and back pain.
Prescribed for	319	Percocet: I was prescribed for kidney stones. definately took the pain away and very high.
		Zoloft: I feel like the antidepressant is used in conjunction with my cymbalta which I am prescribed for both depression and fibromayalgia.
		Celebrex: I was prescribed for knee pain following surgery for torn muniscus.

^a^Consumer comments are shown exactly as they appeared on the WebMD site.

**Table 5:** Frequency of common textual patterns after removing known indications and adverse drug effects.

Pattern	Count	Example drugs and comments^a^
I use * for	171	Flector: it’s not so bad. I use them for stress headaches only if I have a mild headache
		Hydroxyzine: I use this drug for itching attacks and it works fast and effective for me.
		Elavil: I use this medication for restless leg syndrom
I use it for	23	Promethazine: I use it for gastroparesis.i also use it for sleep 4 or 5 times a month
		Amitriptyline: I use it for ic
		Seroquel: I m in love with seroquel its amazing! I use it for sleep and I wake up refreshed
It helps with	72	Neurontin: it helps with numbness in my legs and arms
		Neurontin: I was diagnosed with rsd in from a fall on the ice. It helps with controlling the pain;
		Seroquel: although it helps with my depression I have gained over 50lbs
It help with	6	Oxycontin: it help with muscle spasms
		Hydrocodone-acetaminophen: it is ok I think and it help with my back pian.
		Neurontin: I had drop foot and much pain. It help with the pain along with the 3 epidurals I receiveed in my spine.
I take it	729	Methadone: I take it for chronic pain it helps alot
		Pristiq: I take it for depression and ptsd as well as for chronic pain from failed cervical fusion.
		Zoloft: I have taken it for three years almost and when I take it my depression worsens rather in the summer when I wouldnt take it I was the happiest
I take it for	48	Percocet: I take it for pain after a shoulder surgery and it works
		Buspar: I take it for stress.
		Effexor: I take it for depression.
It works for	155	Pristiq: I do not think it works for me makes me very consipated and I think it makes the back of my legs hurt in the muscle part.
		Metformin: I take it before bed no sideeffect so for taking one month hope it works for me yes I am scared
		Flexeril: back problems healed up then came right back. overall it works for a little while.
Useful for	13	Effexor: I have been reading the reviews of this med. I have been using it for 1.5 yrs and has been very useful for my depression.
		Hydrocodone-acetaminophen: this med. is useful for short term relief of pain.
		Ultram: this med has been very useful for my hip and back pain.
Prescribed for	0	…

^a^Consumer comments are shown exactly as they appeared on the WebMD site.

**Table 6:** Example comments suggesting the possibility of drug repurposing.

Medication	Indication	Adverse effect	Patient comments^a^
Methadone	Dry cough, drug withdrawal syndrome, opioid type drug dependence, and pain	Amenorrhea, phlebitis, sneezing, suffering, withdrawn, hypomagnesemia, urticaria, rhinorrhea, fever, spasm, …	I use this for diabetic neuopathy. Works well with very little side effects.
Elavil	Depression, chronic pain, irritable bowel syndrome, sleep disorders, diabetic neuropathy, agitation and insomnia, and migraine prophylaxis	None in SIDER	elavil is an old school antidepressant that is now considered a dirty drug because of its undesired side effects. one of the unintended side effects is to relax the skeletal muscle tissue. I use elavil off label to treat my tmj
Spironolactone	Low-renin hypertension, hypokalemia, and Conn syndrome	Hyperkalemia, amenorrhea, urticaria, epidermal necrolysis, anaphylaxis, fever, toxic epidermal necrolysis, lethargy, nausea, …	I use it for acne. go figure it works
Strattera	Attention-deficit/hyperactivity disorder, alone or in combination with behavioral treatment	None in SIDER	I was prescribed this medication for slight adhd with off label anxiety help.
Viibryd	Acute episodes of major depression	None in SIDER	It even helps my migraines somewhat (maybe it will be off label in the future for migraine prophylaxis)

^a^Consumer comments are shown exactly as they appeared on the WebMD site.

## Discussion

### Comparison of MetaMap Versus Dictionary-Based Approach

MetaMap is a sophisticated tool that uses natural language processing and machine learning methods; thus, it is more accurate than the dictionary-based approach. MetaMap, to some extent, tackled some general concerns such as disambiguation, misspelling, and word normalization, but none of these is addressed in the dictionary-based approach. For example, in the phrase “My stomach and back hurts to sit, lay down, or stand,” the dictionary-based approach would tag “down” as a disease because of overlap with the “genetic disorder down syndrome.” As Table 2 shows, MetaMap recognized about three times the number of disease names than the dictionary-based approach. The main reason for this difference is word normalization in MetaMap. The dictionary-based approach is limited by its requirement for exact matches—for example, a dictionary that contains only “dizzy” would not detect “dizziness” as a relevant word. In contrast, MetaMap uses stemming and lemmatization to normalize words. The main advantage of dictionary-based mapping over MetaMap is speed (the dictionary-based approach is considerably faster).

### Using Patient Comments for Drug Repurposing

The reviews commonly described general disorders such as pain, itching, and headache. This is expected because comments usually are not authored by medical experts. We observed that patients tend to report adverse drug events instead of beneficial effects, as some of the previous studies reported a similar trend [24]. For example, in the corpus provided by Leaman et al [24], they annotated 157 beneficial effects in 3600 posts, while they found 1260 adverse drug events. Nevertheless, some patient comments contain beneficial effects of medication, which makes social media a useful resource for drug repurposing. This imbalance distribution makes identifying beneficial effects more difficult, especially for training a classifier. Our results (see Tables 4,5, and 6) suggest that an effective approach for this task is to recognize the textual patterns that people used to report beneficial effects (eg, “I use [*drug*] for [*disease*]”). For example, in a review of Viibryd *,* a user mentioned, “It even helps my migraines somewhat,” clearly noting a beneficial effect of the drug, which could be captured by our rule-based system. Similar to other computational drug repurposing approaches, these findings need to be reviewed manually by experts and then confirmed or rejected by laboratory tests or clinical trials. But as these reviews provided by non-expert users, compared to other drug repurposing studies, which use resources provided or generated by experts such as clinical data or biomedical literature, our findings need more validation before going through clinical trials or laboratory tests.

### Limitations

We acknowledge some limitations to this study. Analysis of the patient comments, which are written in an informal manner, obviously needs a system that can handle spelling and grammatical errors. Our current implementation does not address these issues.

Our system covered only simple textual patterns, although examples in Tables 4 and 5 highlight the need to decode complex patterns. A simple pattern-matching system obviously is insufficient for a statement such as “I use it for nose allergies and it does not clear up my nostrils.” A system should be able to handle negation and coreference.

Another limitation of this study was that comments originated from only one forum. Other social media sites such as Yahoo! Answers, PatientsLikeMe [38], and even Twitter have similar information, which can be studied and added to our corpus. In addition, using only one resource for known side effects and one for indication was another limitation. In Table 3, there are several known indications and adverse drug events, which highlight this limitation.

In this study, we were not able to evaluate our system precisely and provide common performance metrics because of the lack of an annotated corpus. As future work, we plan to annotate a corpus of comments from various forums, to allow us to explore this valuable resource extensively and implement and evaluate different approaches.

### Conclusion

We assessed the feasibility of using social media to identify drug-repurposing candidates. After collecting patient reviews of medications from WebMD, we used dictionary-based and MetaMap approaches to identify disorders mentioned in the reviews. Reviews describing known indications or known adverse drug events were excluded, and the remaining reviews were searched for textual patterns commonly used to report beneficial effects. Although the most commonly reported disorders were nonspecific (eg, pain, itching, headache), we nevertheless showed that consumer comments contain beneficial effects of medication and have the potential to be used for drug repurposing. Our textual patterns were able to capture some beneficial effects, but there is a need for a more complex and sophisticated system to identify beneficial effects in social media.

## Abbreviations

NER: named entity recognition
SIDER: SIDe Effect Resource
UMLS: Unified Medical Language System
